# Lnc007185570.1–pal-miR-3120-3p–*ppp3cb* Axis Regulating the Osteogenic Differentiation and Intermuscular Bone Formation in *Megalobrama amblycephala* via the Wnt Signaling Pathway

**DOI:** 10.3390/biology15151302

**Published:** 2026-08-05

**Authors:** Zhengyu Xiao, Jian Jin, Hui Mao, Yizhou Hong, Wenxiu Wang, Qiujie Sun, Xudong Wang, Xuemei Xiong, Shiming Wan, Zexia Gao, Chunhong Nie

**Affiliations:** 1 Key Lab of Freshwater Animal Breeding, Ministry of Agriculture and Rural Affairs/Engineering Research Center of Green Development for Conventional Aquatic Biological Industry in the Yangtze River Economic Belt, Ministry of Education, College of Fisheries, Huazhong Agricultural University, Wuhan 430070, China; xiaozhengyu@webmail.hzau.edu.cn (Z.X.); jinjian@webmail.hzau.edu.cn (J.J.); maohui@webmail.hzau.edu.cn (H.M.); hongyizhou123@webmail.hzau.edu.cn (Y.H.); wangkaka@webmail.hzau.edu.cn (W.W.); qiujiesun@webmail.hzau.edu.cn (Q.S.); wangxudong@webmail.hzau.edu.cn (X.W.); xiongxmbsh@mail.hzau.edu.cn (X.X.); wansm@mail.hzau.edu.cn (S.W.); 2 Hubei Hongshan Laboratory, Wuhan 430070, China; 3 Hubei Province Famous Fish Breeding and Healthy Aquaculture Engineering Technology Research Center, Wuhan 430070, China

**Keywords:** *M. amblycephala*, intermuscular bones, differentiation of osteoblasts, NcRNAs and mRNAs, CeRNA

## Abstract

IBs are mineralized spicules originating from osteoblasts differentiated from tenocytes that can reduce the commercial value of fish and pose potential health risks to consumers if accidentally ingested. *Runx2b* exhibited higher expression in osteoblasts associated with IBs. Knockout of the *runx2b* gene significantly reduced osteoblast numbers and completely inhibited IB formation, confirming its essential role in osteogenic differentiation. However, how non-coding RNAs regulate this process during IB formation remains poorly understood. In this study, whole-transcriptome sequencing was performed to identify ncRNAs and mRNAs in dorsal and tail muscle tissues from six-month-old *M. amblycephala* with IBs (WT group, *n* = 3) and without IBs (Mut group, *n* = 3). A total of 1762 and 1992 differentially expressed (DE) transcripts were identified in dorsal and tail muscle, respectively. Specifically, in dorsal muscle, 408 DE-circular RNAs (circRNAs), 425 DE-long non-coding RNAs (lncRNAs), 171 DE-microRNAs (miRNAs), and 758 DE-messenger RNAs (mRNAs) were detected; in tail muscle, 337 DE-circRNAs, 414 DE-lncRNAs, 222 DE-miRNAs, and 1019 DE-mRNAs were identified. Gene Set Enrichment Analysis (GSEA) revealed that, in the Mut group, key osteogenic signaling pathways, specifically Wnt, MAPK, and TGF-β, were significantly enriched among downregulated genes. Several key IB formation-responsive ceRNAs were identified based on correlation analysis, and the expression and function of the critical regulatory axis, lnc007185570.1–pal-miR-3120-3p–*ppp3cb*, were experimentally validated. Functional assays subsequently demonstrated that this axis modulates osteoblast differentiation via the Wnt signaling pathway. These findings offer new insights into the molecular mechanisms governing osteoblast differentiation during IB formation from the perspective of ceRNA regulatory networks.

## Simple Summary

Intermuscular bones (IBs) compromise the economic value and food safety of fish products. This study compared the expression profiles of non-coding RNAs (ncRNAs) and mRNAs in dorsal and tail muscle tissues from six-month-old *Megalobrama amblycephala* (*M. amblycephala*) with IBs (wild type, WT) and without IBs (mutant, Mut). Our results revealed that loss of IBs was associated with reduced osteoblast differentiation and the downregulation of key bone-related pathways, including Wnt, MAPK, and TGF-β. Furthermore, we identified a competing endogenous RNA (ceRNA) regulatory axis, lnc007185570.1–pal-miR-3120-3p–*ppp3cb*, and demonstrated that it modulates osteoblast differentiation via the Wnt pathway. Collectively, these findings provide novel insights into the molecular mechanisms underlying IB formation.

## 1. Introduction

Intermuscular bones (IBs) are slender, spicule-like structures located within the muscle tissues flanking the vertebral column that originate from myoseptal tendons in teleost fishes [1,2]. In aquaculture, the presence of IBs is primarily associated with processing difficulties, reduced market value, and elevated consumer safety concerns due to the risk of ingestion-related injury [3,4]. Since 2015, researchers’ attention has shifted to the molecular mechanism of IB formation and growth with high-throughput sequencing technologies [5,6]. Single-cell RNA sequencing (scRNA-seq) analysis has demonstrated that IB formation is mediated by a core tendon-osteoblast lineage, in which tendon progenitor cells undergo osteogenic differentiation to form IBs. Moreover, the absence of *runx2b* was found to entirely block IB development [7]. The scRNA data also indicated that osteoblasts are almost completely lacking in *runx2b^−/−^* mutants [7]. However, the exact mechanism underlying osteoblast differentiation remains poorly understood.

Accumulating evidence demonstrates that ncRNAs—including lncRNAs, circRNAs, and miRNAs—play fundamental roles in bone development and homeostasis [5,8]. In skeletal development in animals, ncRNAs serve as critical regulators. For instance, lncRNA LOC100506178 functions as a ceRNA that competitively binds miR-214-5p, thereby relieving the inhibition of *BMP2* and promoting osteogenic differentiation in human bone marrow-derived mesenchymal stem cells (hBMSCs) [9]. Similarly, lncRNA H19 enhances bone formation and angiogenesis in cystathionine β-synthase heterozygous mice by regulating the ANGPT1/TIE2-nitric oxide signaling axis [10]. Nevertheless, research examining how ncRNAs regulate the development of IBs remains limited, with reports restricted so far to *Cyprinus carpio*, *Megalobrama amblycephala*, and *Danio rerio* [5,11,12,13,14]. MSTRG.13175.1 may influence osteogenic differentiation in zebrafish by modulating *sp7* expression through its interaction with dre-miR-2189 [14]. Additionally, in three-year-old *M. amblycephala*, LNC017705 serves as a ceRNA by binding to miR-24a-3p, which in turn regulates *Zip1* expression and inhibits both osteoblast differentiation and calcium deposition, in contrast to the patterns observed in one-year-old individuals [12]. However, several key questions regarding ncRNAs in *M. amblycephala* remain unanswered: the expression profiles of ncRNAs in fish with and without IBs have not been systematically characterized; the regulatory roles of ncRNAs in osteogenic differentiation during IB formation are largely unknown; and whether a ceRNA network governs osteoblast differentiation remains to be elucidated.

The cyprinid fish *M. amblycephala*, which naturally develops IBs, serves as an established model for investigating the molecular regulation of IB development. An *M. amblycephala* strain lacking IBs was previously generated by disrupting *runx2b* via genome editing [15]. In this study, we performed whole-transcriptome sequencing to comprehensively profile ncRNAs and mRNAs in the dorsal and tail muscle tissues of six-month-old *M. amblycephala* with IBs (WT) and without IBs (Mut). Differentially expressed circRNAs, miRNAs, lncRNAs, and mRNAs were identified and subjected to systematic screening and functional enrichment analyses. Subsequently, an IB formation-associated ceRNA regulatory network comprising these circRNAs, miRNAs, lncRNAs, and mRNAs was constructed. Through in vitro experiments, the expression and function of a key ceRNA regulatory axis (lnc007185570.1-pal-miR-3120-3p-*ppp3cb*), which modulates osteoblast differentiation through the Wnt signaling pathway, were validated. Previous studies have identified several key protein-coding genes associated with IB formation in fish and have demonstrated that signaling pathways such as BMP and TGF-β are involved in osteogenic differentiation during IB development [7,12,14]. Despite these advances, the roles of ncRNAs in IB formation remain largely uncharacterized, particularly those of lncRNAs and their ceRNA regulatory networks. This study provides the first systematic characterization of ncRNA expression profiles in *M. amblycephala* with and without IBs, filling a critical gap in our understanding of the molecular mechanisms underlying IB formation. The identified ceRNA network and key regulatory axis offer promising molecular targets for the marker-assisted selection of strains without IBs, which holds significant potential for improving flesh quality and commercial value in aquaculture.

## 2. Experimental Section

### 2.1. Experimental Materials

Using CRISPR-Cas9 gene editing technology, our laboratory generated a *runx2b* knockout strain of *M. amblycephala* that completely lacks IBs (Figure S1A,B) [15]. Dorsal and tail muscle samples were randomly selected from six-month-old *M. amblycephala* with IBs (WT, *n* = 3) and without IBs (Mut, *n* = 3), respectively, yielding a total of 12 samples across two tissues and two groups. All fish were reared under identical conditions to minimize environmental variation. Three biological replicates per group were employed, which is a widely accepted standard for RNA-seq studies in teleost fish and provides adequate statistical power for differential expression analysis given the homogeneous genetic background of the experimental animals. Tissue specimens were rapidly frozen in liquid nitrogen and sent to Zhenyue Biotechnology Co., Ltd. (Wuhan, China). for whole-transcriptome RNA sequencing. Ethical approval for all procedures was authorized by the Huazhong Agricultural University’s Institutional Animal Care and Use Committee (21 March 2023, Approval No. HZAUFI-2023-0013).

### 2.2. Library Preparation and Sequencing

Total RNA extraction was performed with RNAiso Plus Reagent (Cat# 9109, Takara, Kusatsu, Japan) following the manufacturer’s instructions. The quality, yield, and integrity of the isolated RNA were assessed using a NanoDrop 2000 spectrophotometer (Thermo Fisher Scientific, Shanghai, China) and an Agilent 2100 Bioanalyzer (Agilent Technologies, Santa Clara, CA, USA). After passing quality control, construction of the RNA-seq cDNA library was initiated. RNA sequencing libraries were constructed through a standardized workflow: sequence-specific rRNA probes were used to selectively remove rRNA from total RNA, enriching for non-ribosomal transcripts; the rRNA-depleted RNA was then randomly fragmented using Fragmentation Buffer to generate appropriately sized fragments for sequencing. Subsequently, random primers were employed to synthesize the first strand of cDNA using the fragmented RNA as a template, followed by RNase H-mediated degradation of the RNA strand and second-strand cDNA synthesis with dNTPs as substrates, and the resulting double-stranded cDNA (ds-cDNA) was purified to remove reaction byproducts. The purified ds-cDNA underwent end repair, A-tailing, and ligation of sequencing adapters to enable Illumina-compatible sequencing, after which AMPure XP beads (Cat# A63881, Beckman Coulter, Brea, CA, USA) were used to select target-sized fragments for uniform insert length distribution. The uracil-containing second cDNA strand (synthesized from dUTP during second-strand synthesis, if applicable) was specifically degraded using the USER enzyme to preserve strand specificity (if intended), and PCR amplification was finally performed to generate the final cDNA library with sufficient yield and complexity for sequencing. Following library construction, initial quantification was conducted with a Qubit 3.0 fluorometer (Invitrogen, Carlsbad, CA, USA) to confirm concentrations > 1 ng/µL. The size distribution was evaluated via the Qsep400 high-throughput analysis system (BiOptic Inc., New Taipei City, Taiwan, China). Libraries meeting the predetermined size criteria were subsequently quantified by quantitative PCR (qPCR) to determine effective concentrations (>2 nM). Libraries that passed quality checks were processed through paired-end 150 bp sequencing using the Illumina NovaSeq 6000 platform (Illumina, San Diego, CA, USA).

The small RNA library was constructed at Zhenyue Bio following standardized protocols. Total RNA was ligated with 5′ and 3′ adapters to generate adapter-tagged single-stranded RNA, which was reverse-transcribed into RNA/DNA hybrid chains. The hybrids underwent PCR amplification and purification by PAGE gel electrophoresis to produce the small RNA-seq cDNA library, which was subsequently sequenced on an SE50 platform (Illumina, San Diego, CA, USA). Detailed library construction protocols and sequencing parameters are available on the official website of Wuhan Zhenyue Biotechnology Co., Ltd. (Wuhan, China).

### 2.3. Transcriptome Assembly and Annotation of ncRNAs and mRNAs

Following RNA-seq cDNA library sequencing, raw cDNA library reads underwent quality filtering and adapter removal with fastp v0.23.2. The cleaned data were subsequently analyzed using the bioinformatics pipeline supplied by Zhenyue Biotechnology. Clean data were obtained after filtering, followed by alignment to the designated *M. amblycephala* reference genome (GenBank Assembly accession: GCA_018812025.1; available at NCBI, https://www.ncbi.nlm.nih.gov/assembly/GCA_018812025.1, accessed on 15 March 2026) to generate mapped data for subsequent analyses of mRNAs, lncRNAs, and circRNAs.

Transcript assembly of the mapped reads was performed using StringTie. The assembled transcripts were then classified by comparative annotation with the reference genome annotation using gffcompare (v0.11.2). Based on the classification results, transcripts with class_code “i”, “x”, “u”, or “o” that met the criteria of length ≥ 200 bp and ≥2 exons were selected as candidate novel lncRNAs.

Four tools were used for coding potential prediction and protein domain annotation: LGC, CPC2, PLEK, and Pfam. Only transcripts predicted to be non-coding by all four tools (intersection of results) were retained as novel lncRNAs. The identified long non-coding RNAs were named and annotated in accordance with the guidelines established by the HUGO Gene Nomenclature Committee (HGNC).

For expression quantification, StringTie (v2.2.3) was employed to perform gene- and transcript-level quantification separately for known mRNAs and lncRNAs (including both known and novel lncRNAs). Expression matrices of read counts, FPKM, and TPM were generated for downstream analysis.

Two widely used circRNA identification tools, Find_circ (v1.2) and CIRI (v2.0.6), were employed for circRNA detection. CircRNAs identified by both tools (intersection of results) were considered high-confidence candidates. The Find_circ algorithm operates on the following principle: for unmapped reads, a 20-nt anchor sequence is extracted from each end, and the anchor pair is re-aligned to the reference genome. If the 5′ end aligns to the reference (positions A3–A4) and the 3′ end aligns to the upstream region of this locus (positions A1–A2), with a canonical GT-AG splice junction between A2 and A3, the read is classified as a candidate circRNA. Finally, candidate circRNAs with a read count ≥ 2 were defined as validated circRNAs for subsequent analysis.

For small RNA-seq cDNA library data, initial quality control and filtering were performed using fastp (v0.20.1). The resulting high-quality data were then statistically analyzed, providing the foundation for subsequent bioinformatics investigations. The length distribution profile of sRNAs facilitated classification: miRNAs predominantly ranged from 21 to 22 nt in length, while siRNAs showed a peak at 24 nt. Filtered reads were mapped to Rfam (v14.9) and Repbase (v24.01) databases using Bowtie2 (v2.4.5) to eliminate rRNAs, tRNAs, snRNAs, snoRNAs, other annotated ncRNAs, and repetitive elements. The unmapped reads were retained for later miRNA identification. The unmapped reads were subsequently aligned to the reference genome using HISAT2 (v2.2.1). Alignment quality was evaluated with Qualimap (v2.2.2) to confirm data reliability for downstream analysis. Detailed bioinformatics analysis pipelines, including data preprocessing, alignment, and quantification, are available on the official website of Wuhan Zhenyue Biotechnology Co., Ltd.

### 2.4. Identification and Analysis of circRNAs, lncRNAs, miRNAs, and mRNAs

Differential expression analysis of circRNAs, lncRNAs, miRNAs, and mRNAs was performed using DESeq (v1.46.0), with thresholds set at a *p*-value < 0.05 and |log2(fold change)| > 1. Hierarchical clustering was subsequently carried out to visualize expression profiles in both WT and Mut groups. To explore the functional roles of differentially expressed genes (DEGs), Gene Ontology (GO) and Kyoto Encyclopedia of Genes and Genomes (KEGG) enrichment analyses were performed using clusterProfiler (v4.0) in R. Enrichment significance was determined using a hypergeometric test with Benjamini–Hochberg correction for multiple comparisons, and terms with an adjusted *p*-value < 0.05 were considered significantly enriched. Additionally, whole-genome Gene Set Enrichment Analysis (GSEA) was conducted on the mRNA transcriptome data using clusterProfiler (v4.0) with GO and KEGG gene sets. Genes were ranked by fold change for GSEA input, and gene sets with a normalized enrichment score |(NES)| > 1, a nominal *p*-value < 0.05, and a false discovery rate (FDR) < 0.25 were considered significantly enriched.

### 2.5. Construction of circRNA/lncRNA-miRNA-mRNA Regulatory Networks

The ceRNA interaction analysis was conducted using a co-expression-based approach, in which molecular pairs with targeting relationships were identified according to their correlation patterns across multiple samples. Specifically, miRanda (v0.10.22) was used to predict miRNA–target interactions between ncRNAs and mRNAs, with pairs scoring below 140 excluded from further analysis. To evaluate potential regulatory relationships, Pearson correlation analysis was performed to quantify the associations between mRNA expression levels and the corresponding lncRNA/circRNA expression profiles. Pairs with a Pearson correlation coefficient greater than 0.7 were retained. Among these, lncRNA–mRNA and circRNA–mRNA pairs that showed positive correlations and shared miRNA response elements (MREs) were subjected to hypergeometric testing to evaluate the statistical significance of their shared miRNAs. Finally, the ceRNA network was constructed by selecting lncRNA–mRNA and circRNA–mRNA pairs that exhibited significant inverse correlations mediated by shared miRNAs. The resulting regulatory relationships among miRNAs, lncRNAs/circRNAs, and mRNAs were visualized using Cytoscape software (version 3.9.1).

### 2.6. Verification of DE-ncRNAs and DE-mRNAs

To validate the RNA-seq expression profiles, real-time quantitative PCR (RT-qPCR) was performed on 15 randomly selected DE-ncRNAs and DE-mRNAs using the QuantStudio™ 6 Flex System (Thermo Fisher Scientific, Waltham, MA, USA). The candidate genes were selected to span the full dynamic range of the observed expression changes. Differences between the WT and Mut groups were assessed using a *t*-test with IBM SPSS software (v26.0). Gene-specific primers were designed using the NCBI Primer-BLAST tool (Table S1).

### 2.7. Isolation and Culture of Osteoblasts from M. amblycephala

Following the osteoblast culture protocol established for zebrafish [16], the experimental procedure was conducted as follows. Six-month-old *M. amblycephala* were immersed in a 0.01% potassium permanganate solution for 30 min. Using a sterile scalpel, the caudal region posterior to the anus was excised. The skin covering the caudal body surface was carefully peeled away, and the muscle tissue surrounding the IBs was removed, leaving the IBs intact. The IBs were subsequently rinsed with PBS buffer (Servicebio, Wuhan, China) supplemented with 5% penicillin–streptomycin (Servicebio, Wuhan, China) to eliminate residual muscle and connective tissue. Next, the IBs were equilibrated twice for 30 min each in M-199 medium (Servicebio, Wuhan, China) containing 5% penicillin–streptomycin. Subsequently, the IBs were cut into small pieces and immersed in carp serum (JINGKEBIO, Hefei, China), after which they were seeded onto the bottom of a culture flask. The culture flask was incubated at 28 °C with 5% CO_2_ for 2–3 h to facilitate initial cell attachment. Complete M-199 medium containing 5% penicillin–streptomycin and 20% FBS (HYCEZMBIO, Wuhan, China) was then added, and the medium was replaced every 48 h (with one-third of the volume exchanged each time). Upon reaching 80% confluency, the cells were dissociated using trypsin (Servicebio, China), counted, and subcultured to establish primary osteoblasts from *M. amblycephala*.

### 2.8. Vector Construction

The full-length coding sequences of *ppp3cb* and lnc007185570.1 were each cloned into the pCS2+ vector to generate overexpression constructs, designated Ad-pCS2+-*ppp3cb* and Ad-pCS2+-lnc007185570.1, respectively. To investigate the interaction between pal-miR-3120-3p and *ppp3cb*, the 3′ untranslated region (UTR) of *ppp3cb* containing the predicted pal-miR-3120-3p binding site was amplified and inserted downstream of the luciferase coding sequence in the pmir-GLO vector (Promega, Madison, WI, USA), yielding the wild-type reporter construct (pmir-*ppp3cb*-wt). A mutant version of the *ppp3cb* 3′ UTR, in which the pal-miR-3120-3p binding site was disrupted by mutating “gcttgt” to “atcctg”, was synthesized and cloned into the same pmir-GLO backbone to generate the mutant reporter construct (pmir-*ppp3cb*-mut). In addition, the region of lnc007185570.1 encompassing the pal-miR-3120-3p binding site was cloned into the pCS2+ vector, producing the construct pCS2+-lnc007185570.1. Furthermore, pal-miR-3120-3p mimics, si-lnc007185570.1, si-pal-miR-3120-3p, and si-*ppp3cb* were synthesized by GenePharma (Shanghai, China). All constructs were verified by sequencing, and the primer sequences used in this study are listed in Table S1.

### 2.9. Luciferase Activity Assay

The dual-luciferase reporter assay was performed in HeLa cells. The use of heterologous mammalian cell lines for target validation is a well-established approach; HeLa cells were selected owing to their high transfection efficiency and the absence of interference from endogenous teleost ncRNAs. When HeLa cells reached 70–80% confluency in 24-well plates, the following transfection groups were set up: (1) pmir-nc (negative control); (2) pmir-nc + pal-miR-3120-3p mimics; (3) pmir-*ppp3cb*-wt + pal-miR-3120-3p-nc; (4) pmir-*ppp3cb*-wt + pal-miR-3120-3p mimics; (5) pmir-*ppp3cb*-mut + pal-miR-3120-3p mimics; (6) pmir-*ppp3cb*-wt + pal-miR-3120-3p mimics + pCS2+-lnc007185570.1-nc; and (7) pmir-*ppp3cb*-wt + pal-miR-3120-3p mimics + pCS2+-lnc007185570.1. All transfections were performed using Lipofectamine™ 2000 (Thermo Fisher Scientific, Waltham, MA, USA) according to the manufacturer’s instructions, and cells were incubated at 37 °C in a 5% CO_2_ atmosphere. At 48 h post-transfection, cells were lysed (*n* = 3), and luciferase activities were measured using a dual-luciferase assay kit (Vazyme, Nanjing, China). Relative luciferase activity was calculated as the ratio of firefly to Renilla luminescence.

### 2.10. Overexpression and Interference Assays

When osteoblasts derived from *M. amblycephala* reached approximately 80% confluency in 12-well plates, a series of functional experiments was carried out. For overexpression experiments, cells were transfected with Ad-nc, Ad-lnc007185570.1, Ad-pal-miR-3120-3p, or the combination Ad-lnc007185570.1+Ad-pal-miR-3120-3p. For knockdown, cells were transfected with Si-nc, Si-lnc007185570.1, Si-pal-miR-3120-3p, or the combination Si-lnc007185570.1+Si-pal-miR-3120-3p. All transfections were performed using Lipofectamine™ 2000 (Cat# 11668019, Thermo Fisher Scientific, Waltham, MA, USA) according to the manufacturer’s protocol.

For Wnt signaling inhibition, cells were transfected with Ad-nc, Si-Wnt, Ad-pal-miR-3120-3p, or the combination Ad-pal-miR-3120-3p+Si-Wnt. For Wnt signaling activation, cells were transfected with Si-nc, Ad-Wnt, Si-pal-miR-3120-3p, or the combination Si-pal-miR-3120-3p+Ad-Wnt. Additionally, the Wnt signaling activator Laduviglusib (Cat# HY-10182, MCE, Monmouth Junction, NJ, USA) and the inhibitor IWR-1 (Cat# HY-12238, MCE, Monmouth Junction, NJ, USA) were used. Both compounds were dissolved in dimethyl sulfoxide (DMSO; Sigma-Aldrich, St. Louis, MO, USA) to prepare 10 mM stock solutions, which were stored at −20 °C in the dark. For treatment, the stock solutions were diluted in culture medium to a final working concentration of 10 μM, with DMSO at a final concentration of <0.1% (*v*/*v*). An equivalent volume of DMSO was added to the vehicle control group. Cells were incubated with the compounds for 12 h prior to downstream analysis. At 48 h post-transfection, total RNA and protein were extracted from the cells (*n* = 3), and RT-qPCR and Western blot analyses were conducted to confirm transfection efficiency and validate successful overexpression (Ad groups) and interference (Si groups).

### 2.11. RT-qPCR

Total RNA was extracted from cells and tissues using RNAiso Plus reagent (Takara, Kusatsu, Japan) following the manufacturer’s protocol. The quality, yield, and integrity of the isolated RNA were assessed using a NanoDrop 2000 spectrophotometer (Thermo Fisher Scientific, Waltham, MA, USA) and 1% agarose gel electrophoresis. Only samples with an OD260/280 ratio between 1.9 and 2.1 and an OD260/230 ratio > 2.0 were considered qualified for subsequent experiments. For mRNA, lncRNA, and circRNA RT-qPCR analysis (before performing circRNA quantification, total RNA was treated with DNase I (Vazyme, Nanjing, China) to remove genomic DNA contamination), complementary DNA (cDNA) was synthesized using the HiScript III 1st Strand cDNA Synthesis Kit (Vazyme, Nanjing, China). For miRNA RT-qPCR analysis, cDNA was prepared with the miRNA 1st Strand cDNA Synthesis Kit (Vazyme, Nanjing, China). RT-qPCR was performed using Hieff^®^ qPCR SYBR Green Master Mix (Yeasen, Shanghai, China) and gene-specific primers. Amplification and detection were conducted on a QuantStudio 6 Flex Real-Time PCR System (Applied Biosystems, ABI, Waltham, MA, USA) (*n* = 3). The relative expression levels of target genes were calculated using the comparative CT method (2^−ΔΔCT^). Detailed information on all primers used in this study is provided in Table S2.

### 2.12. Western Blotting

Total protein was extracted using a universal lysis buffer for Western blotting (AFGL-100, Affinibody, Wuhan, China). Protein concentrations were determined using a BCA assay kit (P0012, Beyotime, Shanghai, China). Samples were denatured at 98 °C for 10 min in loading buffer and then resolved on a 10% PAGE gel (SF10, Affinibody, Wuhan, China). Subsequently, proteins were transferred onto a 0.2 μm PVDF membrane (BR00103, Affinibody, Wuhan, China). Membranes were blocked with 5% skim milk for 2 h, followed by overnight incubation at 4 °C with the corresponding primary antibodies. After washing with TBST, the membranes were incubated with HRP-conjugated secondary antibodies (AS014, ABclonal, Wuhan, China; 1:5000) for 1 h, followed by further TBST washes. Protein bands were visualized using an ECL chemiluminescence detection reagent (BL523A, Biosharp, Beijing, China), and luminescence signals were captured with a Tanon chemiluminescence imager (5200, Tanon, Shanghai, China). The anti-*ppp3cb* primary antibody (13340-1-AP, 1:2000) was obtained from Proteintech (Wuhan, China), and the anti-β-actin antibody (AC038, 1:10,000) was purchased from ABclonal (Wuhan, China). The protein marker was sourced from Sangon Biotech (C610013, Shanghai, China).

### 2.13. Alizarin Red S Staining

*M. amblycephala* osteoblasts were maintained in osteogenic medium containing 0.2% ascorbic acid, 0.01% dexamethasone, and 1% β-glycerophosphate (Sigma, St. Louis, MO, USA) for a period of 21 days. The osteoblasts were then fixed and rinsed with PBS. The fixed cells underwent Alizarin Red S staining (Beyotime, Shanghai, China) for 30 min, followed by thorough rinsing with deionized water. Mineralized nodule formation was visualized under a microscope (SZX16, Olympus, Tokyo, Japan).

### 2.14. Statistical Analysis

Statistical analyses were performed using SPSS (v26.0). For comparisons between two groups, unpaired Student’s *t*-tests were used. For comparisons involving three or more groups, one-way analysis of variance (ANOVA) followed by Tukey’s post hoc test was employed. Graphs were created using GraphPad Prism (v7.0, San Diego, CA, USA). Statistical significance (*p* < 0.05) was denoted by distinct letters, and data were expressed as the mean ± SD unless specified otherwise.

## 3. Results

### 3.1. NcRNAs Identification and Characterization

**Figure 1:**
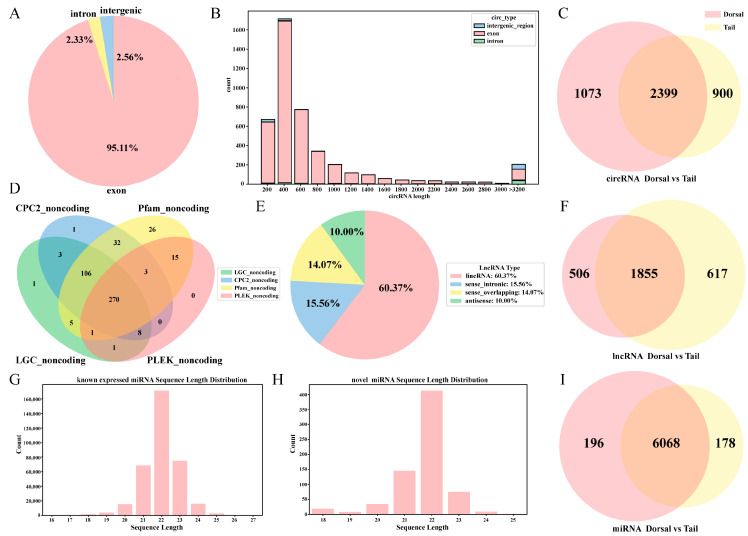
Overview of ncRNA sequencing data of dorsal and tail muscles from *M. amblycephala* strains with IBs (WT) and without IBs (Mut). (**A**) The source of circRNAs; (**B**) The length distribution of circRNAs from all libraries; (**C**) Venn diagram showing shared and unique circRNAs of dorsal and tail muscle; (**D**) Identification of novel lncRNAs based on four algorithms; (**E**) Proportion of novel lncRNAs for each type; (**F**) Venn diagram showing shared and unique lncRNAs of the dorsal and tail; (**G**) Length distribution of known miRNA from all libraries; (**H**) Distribution of lengths for novel miRNA from all libraries; (**I**) Venn diagram showing shared and unique miRNAs between the dorsal and tail muscle.

To investigate the involvement of ncRNAs in osteoblast differentiation, we performed whole-transcriptome sequencing of dorsal and tail muscle tissues from six-month-old *M. amblycephala*, comparing WT and Mut strains. Three biological replicates were analyzed per group. Genomic origin analysis revealed that 95.11% of the identified circRNAs were derived from exonic regions, 2.33% from intronic sequences, and 2.56% from intergenic regions (Figure 1A). Exon-derived circRNAs constituted the predominant category, with their length distribution centered primarily around 400 nucleotides (Figure 1B). Comparative profiling across tissues identified 3472 circRNAs in dorsal muscle and 3299 in tail muscle, of which 2399 were shared between the two tissues (Figure 1C). Furthermore, integrative analysis using four computational algorithms (CPC2, Pfam, LGC, and PLEK) enabled the annotation of 270 novel lncRNAs (Figure 1D). Genomic localization analysis classified 60.37% as lincRNAs (long intergenic non-coding RNAs), 15.56% as sense_intronic, 14.07% as sense overlapping, and 10.00% as antisense lncRNAs (Figure 1E). Tissue-specific profiling demonstrated 2361 lncRNAs in dorsal muscle and 2472 in tail muscle, with 1855 lncRNAs commonly conserved between both tissues (Figure 1F). Length distribution analysis confirmed that both known and novel miRNAs exhibited a predominant size range of 20–24 nucleotides (Figure 1G,H). Comparative profiling identified 6264 miRNAs in dorsal muscle and 6246 in tail muscle, with 6068 miRNAs commonly shared between both tissues (Figure 1I).

### 3.2. DE-ncRNAs Identification and Functional Enrichment Analysis

**Figure 2:**
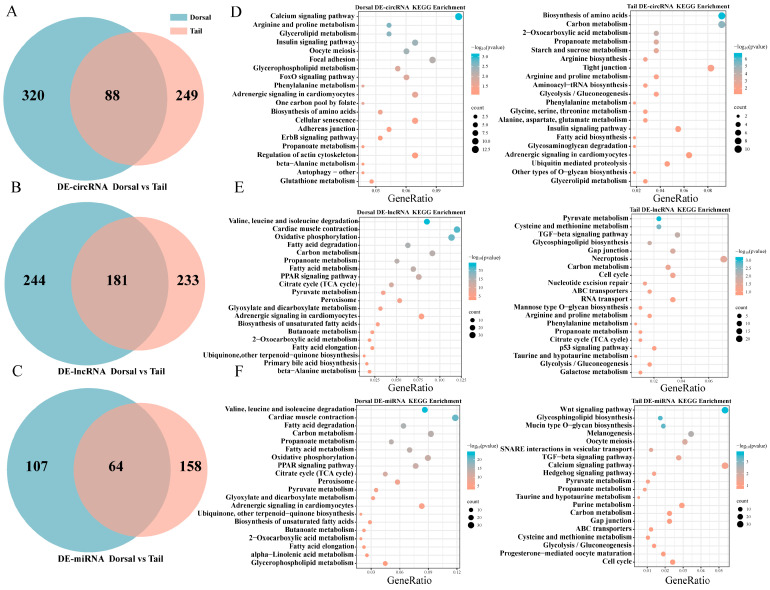
Differential expression and functional enrichment of ncRNAs in dorsal and tail muscles of WT and Mut *M. amblycephala* strains. (**A**–**C**) Venn diagram showing shared and unique DE-circRNAs, DE-lncRNAs, and DE-miRNAs in dorsal and tail muscle of WT vs. Mut; (**D**–**F**) KEGG enrichment analysis of target genes of DE-circRNAs, DE-lncRNAs, and DE-miRNAs in dorsal and tail muscle of WT vs. Mut.

To investigate the expression of ncRNAs between the WT and Mut strains of *M. amblycephala*, differential expression analysis was performed. In the dorsal muscle tissues, 408 DE-circRNAs, 425 DE-lncRNAs, and 171 DE-miRNAs were identified (Figure 2A–C). In the tail muscle, 337 DE-circRNAs (Figure 2A), 414 DE-lncRNAs (Figure 2B), and 222 DE-miRNAs (Figure 2C) were identified. Notably, 88 DE-circRNAs (Figure 2A), 181 DE-lncRNAs (Figure 2B), and 64 DE-miRNAs (Figure 2C) were shared between dorsal and tail muscle tissues. GO analysis revealed that the target genes of DE-circRNAs in both dorsal and tail muscle tissues were primarily associated with muscle development processes (e.g., muscle structure development and muscle organ development) (Figure S2A). DE-lncRNA target genes showed significant enrichment in energy metabolism pathways (e.g., cellular respiration and aerobic respiration) (Figure S2B). Dorsal-specific DE-miRNAs predominantly targeted genes associated with ion channel functions, particularly potassium and cation channel activities. By contrast, tail-muscle-specific DE-miRNAs predominantly targeted genes associated with glycosylation-related modifications and oxidoreductase activity (Figure S2C). KEGG pathway analysis further revealed that target genes of dorsal-muscle-specific DE-circRNAs were significantly enriched in the calcium and ErbB signaling pathways (Figure 2D), including key skeletal development genes such as *mapk1* and *smad2* (Table S3). Conversely, target genes of tail-muscle-specific DE-circRNAs were primarily linked to carbon metabolism, amino acid biosynthesis, and fatty acid biosynthesis (Figure 2D, Table S4). The target genes of DE-lncRNAs in both dorsal and tail muscle tissues were enriched in lipid metabolism pathways, such as fatty acid metabolism and oxidative phosphorylation (Figure 2E; Tables S5 and S6). Dorsal-muscle DE-miRNA targets were significantly enriched in the TGF-β and p53 signaling pathways (Figure 2F), with key skeletal development genes including *bmp7b* and *smad3a* prominently represented (Table S7). Meanwhile, tail-muscle DE-miRNA targets showed significant enrichment in the Wnt, TGF-β, and calcium signaling pathways (Figure 2F), with critical regulators of skeletal development such as *wnt9b*, *mycb*, and *fzd1* being significantly enriched (Table S8).

### 3.3. Differential Gene Expression and Functional Enrichment Analysis

**Figure 3:**
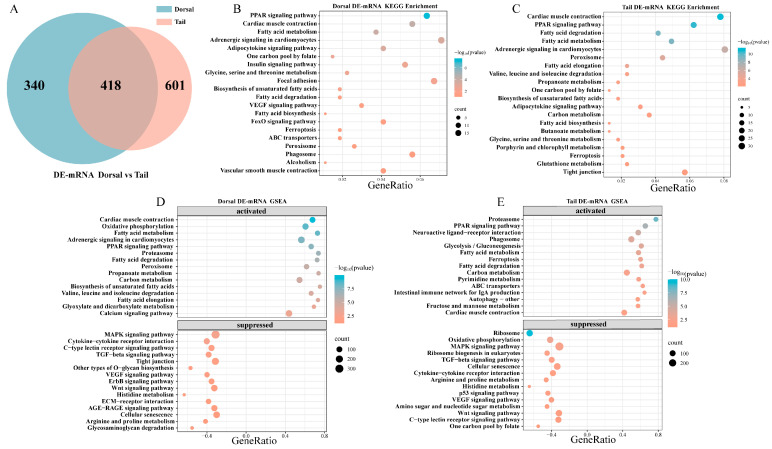
Differential gene expression and functional enrichment analysis in the dorsal and tail muscles of WT and Mut *M. amblycephala* strains. (**A**) Venn diagram showing shared and unique DE-mRNAs in the dorsal and tail of WT vs. Mut; (**B**) KEGG enrichment analysis of DE-mRNAs in the dorsal muscle of WT vs. Mut; (**C**) KEGG enrichment analysis of DE-mRNAs in the tail muscle of WT vs. Mut; (**D**) GSEA of KEGG in the dorsal muscle of WT vs. Mut; (**E**) GSEA of KEGG in the tail muscle of WT vs. Mut.

In the comparative analysis between WT and Mut strains of *M. amblycephala*, 758 DE-mRNAs were identified in dorsal muscle and 1019 DE-mRNAs were identified in tail muscle (Figure 3A), with 418 DE-mRNAs shared between both tissues (Figure 3A). GO analysis revealed significant enrichment in muscle-related biological processes for both dorsal and tail strains, including muscle contraction, skeletal muscle contraction, and cytoskeletal motor activity (Figure S3A,B). KEGG pathway analysis revealed conserved metabolic involvement across strains. DE-mRNA targets from both dorsal and tail muscle displayed enrichment in PPAR signaling, fatty acid metabolism pathways, and cardiac muscle contraction (Figure 3B,C, Tables S9 and S10). Gene Set Enrichment Analysis (GSEA) demonstrated significant suppression of key osteogenic signaling cascades in both dorsal and tail muscle tissues (Figure 3D,E). Comparison of the two tissues revealed shared suppression patterns, suggesting conserved molecular mechanisms underlying IB formation across different muscle regions. The most prominently inhibited pathways were as follows. Wnt signaling pathway (involving *fzd7a*, *smad4*, and *sox17*) is a master regulator of osteoblast differentiation and bone formation. MAPK signaling pathway (including *fgf2*, *fgf4*, and *mapk9*) mediates osteoblast proliferation and differentiation in response to growth factors. TGF-β signaling pathway (featuring *bmp2b*, *bmp15*, and *smad1*) plays critical roles in osteogenic commitment and matrix mineralization (Tables S11 and S12).

### 3.4. Construction of the CeRNA Network Related to IB Formation

**Figure 4:**
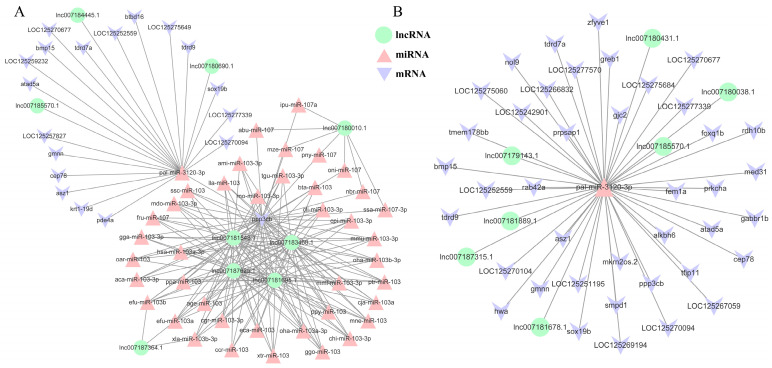
CeRNA network construction in WT and Mut *M. amblycephala*. (**A**) lncRNA-miRNA-mRNA co-expression network in the dorsal muscle of WT and Mut; (**B**) lncRNA-miRNA-mRNA co-expression network in the tail muscle of WT and Mut.

To construct the lncRNA/circRNA-associated ceRNA network, negatively correlated miRNA–mRNA pairs were first screened and visualized. A total of 7745 miRNA–mRNA co-expression pairs were identified in dorsal muscle and 12,932 in tail muscle (Tables S13 and S14). Notably, a conserved miRNA–mRNA interaction, pal-miR-3120-3p–*ppp3cb*, was detected in both dorsal and tail muscle tissues (Pearson correlation coefficient = −0.86, *p* < 0.001). miR-3120 deficiency in humans has been associated with varying degrees of skeletal dysplasia [17], and previous studies in mice have demonstrated that elevated *ppp3cb* expression may inhibit osteoblast differentiation [18]. Using this IB-related core regulatory pair as an anchor, 196 and 241 lncRNA–miRNA–mRNA regulatory pairs were subsequently identified in dorsal and tail muscle, respectively (Figure 4A,B; Tables S15 and S16). The dorsal network comprised 9 lncRNAs, 41 miRNAs, and 19 mRNAs, whereas the tail network consisted of 7 lncRNAs, 1 miRNA, and 40 mRNAs. Additionally, 433 and 160 circRNA-miRNA-mRNA regulatory pairs were identified in dorsal and tail tissues, respectively (Figure S4A,B; Tables S17 and S18). Importantly, a core ceRNA regulatory axis (lnc007185570.1-pal-miR-3120-3p-*ppp3cb*) was identified, which was conserved in both dorsal and tail muscle.

### 3.5. Expression Validation

To validate the expression of DE-ncRNAs and DE-mRNAs, RT-qPCR analysis was performed on a randomly selected subset of 15 differentially expressed transcripts, including 11 DE-ncRNAs and 4 DE-mRNAs. These transcripts were selected to represent a range of expression levels (high, medium, and low abundance) and fold changes (both upregulated and downregulated), covering the major expression patterns observed in the transcriptome data. Compared with the dorsal muscle in WT, *bacb10*, MSTRG.133.2, abu-miR-122, dre-miR-192, dre-miR-7a, and abu-miR-182 were significantly upregulated in Mut, whereas *sox9a*, *bach2a*, *bada*, MSTRG.1085.14, MSTRG.1932.1, circ5959766, circ1522016, circ19503075, and circ33689581 were significantly downregulated. The expression patterns obtained from RT-qPCR showed strong concordance with the RNA-seq data (Figure S5).

### 3.6. Molecular Validation of lnc007185570.1-pal-miR-3120-3p-ppp3cb

**Figure 5:**
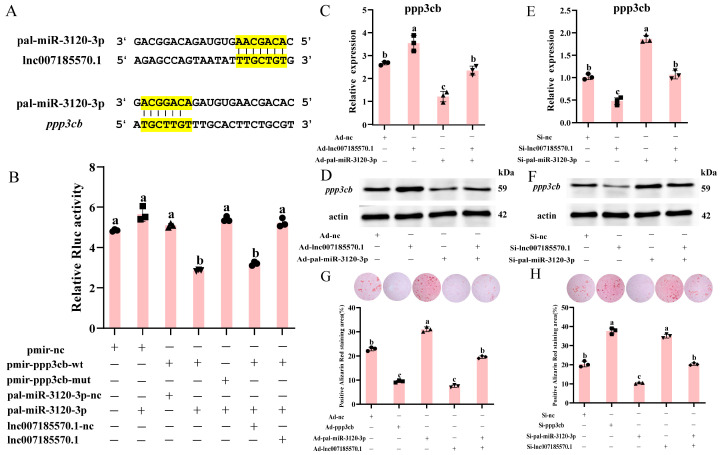
The role of lnc007185570.1-pal-miR-3120-3p-*ppp3cb* pair during osteoblast differentiation. (**A**) Targeting relationship prediction of lnc007185570.1-pal-miR-3120-3p-*ppp3cb* (The yellow highlighted parts are the binding sites); (**B**) Targeting relationship verification of lnc007185570.1-pal-miR-3120-3p-*ppp3cb*; (**C**) RT-qPCR analysis in OBs with Ad-lnc007185570.1 or Ad-pal-miR-3120-3p; (**D**) Western blot analysis in OBs with Ad-lnc007185570.1 or Ad-pal-miR-3120-3p; (**E**) RT-qPCR analysis in OBs with Si-lnc007185570.1 or Si-pal-miR-3120-3p; (**F**) Western blot analysis in OBs with Si-lnc007185570.1 or Si-pal-miR-3120-3p; (**G**,**H**) Alizarin red staining confirming calcification of osteoblasts. The notation used to denote statistical significance is as follows: different letters denote significant differences (*p* < 0.05).

We further investigated the functional mechanism of the conserved ceRNA regulatory axis common to both dorsal and tail muscle tissues in *M. amblycephala*. To verify the targeting relationships among lnc007185570.1-pal-miR-3120-3p-*ppp3cb*, bioinformatic analyses were first performed to identify their binding sites (Figure 5A). Subsequently, these interactions were validated using a dual-luciferase reporter assay in HeLa cells (*n* = 3). The results showed that pal-miR-3120-3p directly repressed *ppp3cb*, resulting in decreased luciferase activity. In contrast, lnc007185570.1 competitively bound pal-miR-3120-3p, thereby relieving the suppression of *ppp3cb* and restoring luciferase activity (Figure 5B).

Next, we investigated the effects of overexpressing the lnc007185570.1-pal-miR-3120-3p-*ppp3cb* axis during osteoblast differentiation in *M. amblycephala*. Overexpression of lnc007185570.1 alone upregulated *ppp3cb* expression, whereas pal-miR-3120-3p overexpression alone downregulated it; co-overexpression of both restored *ppp3cb* levels (Figure 5C). Western blot analysis confirmed these trends at the protein level (Figure 5D).

Complementary loss-of-function experiments yielded consistent patterns. Silencing lnc007185570.1 alone downregulated *ppp3cb*, while inhibition of pal-miR-3120-3p alone upregulated it; co-inhibition restored *ppp3cb* expression (Figure 5E). These changes were also validated at the protein level by Western blotting (Figure 5F).

To assess the functional consequences on osteoblast calcification, alizarin red staining was performed. Overexpression of lnc007185570.1 and *ppp3cb* inhibited osteoblast calcification, whereas co-overexpression with pal-miR-3120-3p partially reversed this suppressive effect (Figure 5G). Conversely, inhibition of lnc007185570.1 and *ppp3cb* promoted calcification, while pal-miR-3120-3p inhibition reduced it, with co-inhibition partially reversing this effect (Figure 5H). Collectively, these data demonstrate that lnc007185570.1 modulates *ppp3cb* via sponging pal-miR-3120-3p, thereby regulating osteoblast calcification.

### 3.7. The lnc007185570.1-pal-miR-3120-3p-ppp3cb Regulates the Differentiation of Osteoblasts via the Wnt Signaling Pathway

**Figure 6:**
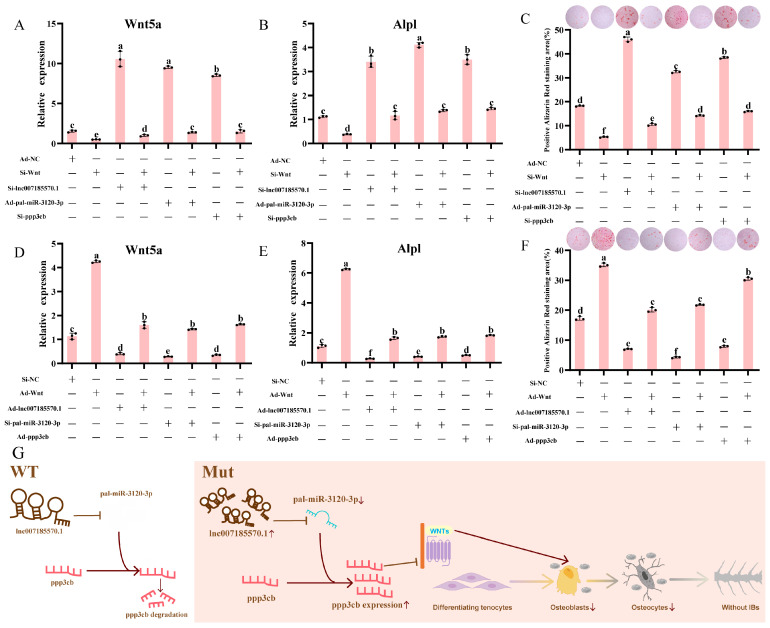
The key ceRNA pair regulates osteoblast differentiation via the Wnt pathway. (**A**,**B**) RT-qPCR analysis detected the expression of *Wnt5a* and *alpl*; (**C**) Alizarin red staining confirmed osteoblast calcification; (**D**,**E**) RT-qPCR detected the expression of *wnt5a* and *alpl*; (**F**) Alizarin red staining verified osteoblast calcification; (**G**) Schematic diagram illustrating how the key ceRNA pair regulates osteoblast differentiation through the Wnt pathway. Statistical significance is denoted by different letters (*p* < 0.05). ↑ indicates an increase; ↓ indicates a decrease.

Previous research has demonstrated the critical involvement of Wnt signaling in osteogenic differentiation [19]. Existing evidence indicates that lnc007185570.1 exerts an inhibitory effect on osteogenic differentiation by sponging pal-miR-3120-3p to upregulate *ppp3cb* expression. Sequencing data revealed that the *ppp3cb* gene was present among the core enriched genes of the Wnt signaling pathway in both dorsal and tail muscle based on GSEA KEGG analysis, leading us to hypothesize that the lnc007185570.1-pal-miR-3120-3p-*ppp3cb* axis modulates osteoblast differentiation in *M. amblycephala* via this pathway. Building on these findings, the relationship with the Wnt signaling pathway was investigated by overexpressing and interfering with individual components of this ceRNA network. Functional analyses revealed that individual treatment with si-lnc007185570.1, ad-pal-miR-3120-3p, or si-*ppp3cb* markedly increased the expression of the Wnt pathway marker gene *wnt5a* and the osteogenic marker gene *alpl* (Figure 6A,B), while also enhancing mineralization capacity (Figure 6C). Notably, these enhancing effects were partially reversed when the Wnt pathway was suppressed using a specific inhibitor (Figure 6A–C). Conversely, individual treatment with ad-lnc007185570.1, si-pal-miR-3120-3p, or ad-*ppp3cb* downregulated the expression of *wnt5a* and *alpl* and reduced the mineralization capacity (Figure 6D–F), and these effects were abolished by a Wnt pathway activator (Figure 6D–F). Collectively, our findings uncover a novel ceRNA regulatory mechanism. In this mechanism, lnc007185570.1 sequesters miR-3120-3p, thereby relieving the inhibition of *ppp3cb* and ultimately suppressing Wnt pathway-mediated differentiation of osteoblasts.

## 4. Discussion

In recent years, significant progress has been made in understanding the molecular mechanisms underlying IB formation and growth [4,20,21]. Advances in high-throughput sequencing and gene editing technologies have enabled the identification of key genes associated with IB counts, including *sp7*, *bmp6*, *runx2b*, *asb14a*, and *asb15b* [7,22,23,24,25]. Among these, *runx2b* and *bmp6* have been successfully applied to generate new strains without IBs in economically important fish species [15,26,27]. While these studies have advanced our understanding of protein-coding genes in IB formation, the upstream regulatory networks governing osteoblast differentiation, particularly the contributions of ncRNAs such as lncRNAs and miRNAs remain largely uncharacterized. Whole-transcriptome sequencing provides a powerful approach for comprehensively profiling all RNA molecules, including ncRNAs, and has been widely used to investigate ceRNA regulatory mechanisms in osteogenic differentiation. In mammals, for example, whole-transcriptome studies have identified key lncRNA–miRNA–mRNA networks regulating osteoblast differentiation in bone marrow mesenchymal stem cells [28,29]. In teleost fish, previous studies have characterized the expression dynamics of miRNAs, mRNAs, and lncRNAs during IB development in *M. amblycephala* [11,13]. Building on these foundations, the present study employed comparative whole-transcriptome sequencing of dorsal and tail muscle tissues from *M. amblycephala* with and without IBs to elucidate the ncRNA-mediated regulatory mechanisms underlying osteoblast differentiation during IB formation. This approach provides novel insights into IB development from the perspective of ncRNA regulation.

In this study, whole-transcriptome sequencing analysis was performed on dorsal and tail tissues from *M. amblycephala* with and without IBs. We compared WT strains with IBs against Mut strains lacking IBs. Differential expression analysis revealed 408 circRNAs, 425 lncRNAs, 171 miRNAs, and 758 mRNAs with significant expression changes in dorsal muscle between the WT and Mut groups. In the tail muscle tissues, 414 DE-lncRNAs, 337 DE-circRNAs, 222 DE-miRNAs, and 1019 DE-mRNAs were identified in the WT-vs-Mut comparison. Notably, 181 DE-lncRNAs, 88 DE-circRNAs, 64 DE-miRNAs and 418 DE-mRNAs were shared between dorsal and tail muscle, suggesting conserved regulatory mechanisms underlying IB formation across different muscle regions. GSEA revealed significant suppression of key osteogenic signaling cascades in both dorsal and tail muscle tissues (Figure 3D,E). The most prominently inhibited pathways included Wnt signaling, MAPK signaling, and TGF-β signaling—all of which have well-established roles in osteoblast differentiation and skeletal development. Specifically, the p38 MAPK pathway is essential for regulating osteoblast differentiation and skeletogenesis [30,31]; the TGF-β signaling pathway plays fundamental roles in osteoblast and chondrocyte lineage commitment, as well as skeletal development and homeostasis [32,33]; and the Wnt signaling pathway serves as a critical regulator of skeletal development and homeostasis [34,35,36]. Notably, the Wnt signaling pathway showed a suppressive trend in both dorsal and tail tissues (dorsal: NES = −1.31, *p* = 0.06; tail: NES = −1.36, *p* = 0.036). Although the *p*-value in dorsal muscle did not reach the conventional significance threshold of *p* < 0.05, the consistently negative normalized enrichment scores (NESs) across both tissues indicate an inhibitory tendency of Wnt signaling in the strain without IBs. To place our findings within the broader framework of cyprinid fish research, we compared signaling pathways associated with IB formation across three representative species: grass carp (*Ctenopharyngodon idella*), zebrafish, and *M. amblycephala*. In zebrafish, differentially expressed genes following *runx2b* knockout were primarily enriched in the TGF-β and Wnt signaling pathways [14]. In grass carp, transcriptomic analysis identified Wnt and MAPK signaling as key pathways involved in IB-related osteogenic differentiation [37]. Notably, in the present study, Wnt, MAPK, and TGF-β signaling were all significantly suppressed in the mutant strain of *M. amblycephala*. This cross-species comparison reveals that the Wnt signaling pathway is consistently associated with IB formation across all three cyprinid species examined, suggesting that Wnt signaling represents a conserved molecular mechanism regulating osteoblast differentiation during IB development. However, while previous studies in zebrafish and grass carp have primarily focused on protein-coding genes, the regulatory roles of ncRNAs within these pathways remain unexplored. This study provides valuable insights into the roles of both ncRNAs and mRNAs in IB development in *M. amblycephala* at the whole-transcriptome level and lays a foundation for comparative studies aimed at elucidating the general molecular mechanisms underlying IB formation in teleost fish.

CeRNAs regulate gene expression through miRNA sponging during the bone formation process in mammals and fish [38,39,40,41]. A representative case involves lncRNA LOC100506178, which enhances osteogenic differentiation in hBMSCs by sequestering miR-214-5p and upregulating *bmp2* expression [9]. Likewise, lncRNA LncMSTRG25 promotes osteogenesis through miR-939-5p sponging, inducing *pax8* transcriptional activation in hBMSCs [42]. In teleosts, lncRNA LNC017705 functions as a ceRNA by competitively binding miR-24a-3p, thereby modulating zinc transporter *zip1* expression and suppressing osteoblast differentiation [13]. Furthermore, MSTRG.13175.1 regulates *sp7* expression by binding to dre-miR-2189, promoting osteogenic differentiation [14]. In the present study, we identified a conserved lnc007185570.1–pal-miR-3120-3p–*ppp3cb* axis in both dorsal and tail muscles (Figure 4). Notably, miR-3120 deficiency in humans leads to varying degrees of skeletal dysplasia [17], underscoring its functional role in skeletal phenotype regulation. In mice, prior studies demonstrated that *DEC1* promotes osteogenic differentiation, whereas *DEC1* deficiency significantly upregulates *ppp3cb* expression [18], suggesting that elevated *ppp3cb* expression may inhibit osteoblast differentiation. Our in vitro experiments further revealed that lnc007185570.1 acts as a molecular sponge for pal-miR-3120-3p, thereby alleviating its suppression of *ppp3cb*. Upregulated *ppp3cb* expression inhibits osteoblast differentiation (Figure 5). This ceRNA regulatory axis represents a novel mechanism underlying IB formation, linking non-coding RNA regulation to osteogenic differentiation in *M. amblycephala*.

The Wnt signaling pathway is critically involved in mammalian bone regeneration and osteoblast differentiation processes [43,44,45], and miRNAs regulate osteoblast differentiation by targeting the Wnt signaling pathway [46]. Wnt5a is a significant marker of the Wnt pathway and promotes osteoblast differentiation [47]. Mouse studies have demonstrated that impaired *wnt5a* signaling inhibits the normal fracture healing process [48], while rat studies have revealed that *wnt5a* participates in BMP-2-mediated osteoblast differentiation through non-Smad-dependent pathways [49]. Previous study have indicated that the Wnt pathway plays an important role during bone development in zebrafish [50,51]. Consistent with these findings, our functional experiments demonstrated that pal-miR-3120-3p significantly upregulates the expression of the Wnt pathway marker *wnt5a* and the osteogenic marker *alpl*, while enhancing osteoblasts differentiation (Figure 6). Wnt pathway inhibition partially reverses these effects, suggesting that the lnc007185570.1-pal-miR-3120-3p-*ppp3cb* axis regulates IB formation in *M. amblycephala* via Wnt signaling. These findings reveal the core regulatory roles of both ceRNA networks and Wnt signaling in osteogenic differentiation during IIB formation This study not only advances our understanding of the molecular mechanisms underlying IB development but also provides promising molecular targets for the genetic breeding of cyprinid fish without IBs. However, several limitations of this study should be acknowledged. First, our transcriptomic analysis was based on bulk RNA sequencing of whole muscle tissues, which cannot distinguish cell-type-specific expression patterns. Future studies employing single-cell RNA sequencing would provide higher-resolution insights into the specific cell types involved in IB formation and their regulatory networks. Second, the functional validation of the ceRNA axis was performed primarily in vitro using cultured osteoblasts; in vivo validation using gene editing technologies (e.g., CRISPR/Cas9 knockout of lnc007185570.1) would further confirm the physiological relevance of this regulatory axis. Third, while we identified the lnc007185570.1–pal-miR-3120-3p–*ppp3cb* axis as a key regulator, additional ceRNA pairs likely contribute to the complex regulatory network governing IB formation. Future studies should systematically characterize more ceRNA axes and their interactions to construct a comprehensive regulatory map. Finally, our study was conducted in *M. amblycephala*; cross-species validation in other cyprinid fish would help determine whether the identified ceRNA regulatory mechanism is conserved across species and assess its broader applicability in aquaculture breeding.

## 5. Conclusions

In summary, through whole-transcriptome sequencing of six-month-old *M. amblycephala* strains with and without IBs, we characterized divergent transcriptomic landscapes associated with IB formation. GSEA revealed significant suppression of key osteogenic signaling pathways, including Wnt, MAPK, and TGF-β signaling, in the strain without IBs. Construction and functional analysis of the ceRNA network further identified the lnc007185570.1–pal-miR-3120-3p–*ppp3cb* axis as a critical regulator of osteoblast differentiation. Mechanistically, lnc007185570.1 functions as a molecular sponge for pal-miR-3120-3p, thereby de-repressing *ppp3cb* expression and modulating downstream Wnt signaling to regulate osteoblast calcification. These findings uncover a previously unrecognized ceRNA-mediated regulatory mechanism underlying IB formation. From a theoretical perspective, this study expands our understanding of non-coding RNA functions in teleost IB development and provides a valuable resource for investigating the conserved regulatory mechanisms of IB formation across cyprinid species. From a practical breeding standpoint, the identified ceRNA axis, particularly lnc007185570.1 and pal-miR-3120-3p offers promising molecular markers and potential gene-editing targets for accelerating the breeding of strains without IBs in economically important cyprinid fish. Nevertheless, further functional validation in vivo and investigation of additional ceRNA axes are needed to fully elucidate the comprehensive regulatory network governing IB formation.

## Data Availability

Raw data of the dataset PRJNA1346052 are available on the SRA database accessed on 16 October 2025 (https://www.ncbi.nlm.nih.gov/bioproject/PRJNA1346052/).

## Supplementary Materials

The following supporting information can be downloaded at https://www.mdpi.com/article/10.3390/biology15151302/s1, Figure S1: IBs phenotypes in WT and Mut individuals of M. amblycephala. (A) X-ray scanning at six-month-old in WT, (B) X-ray scanning at six-month-old in Mut; Figure S2: GO enrichment analysis of DE-ncRNAs in dorsal and tail muscle from WT-vs-Mut. (A) GO enrichment analysis of DE-circRNAs target genes in dorsal and tail from WT-vs-Mut, (B) GO enrichment analysis of DE-lncRNAs target genes in dorsal and tail from WT-vs-Mut, (C) GO enrichment analysis of DE-miRNAs target genes in dorsal and tail from WT-vs-Mut; Figure S3: GO enrichment analysis of DE-mRNAs in dorsal and tail muscle from WT-vs-Mut. (A) GO enrichment analysis of DE-mRNAs target genes in dorsal from WT-vs-Mut, (B) GO enrichment analysis of DE-mRNAs target genes in tail from WT-vs-Mut; Figure S4: ceRNA network construction in *M. amblycephala* from WT-vs-Mut. (A) circRNA-miRNA-mRNA co-expression network in dorsal, (B) circRNA-miRNA-mRNA co-expression network in tail; Figure S5: Validation of transcriptome sequencing. * The x-axis represents RNA names, whereas the left y-axis denotes the ratio of mean expression values (Mut vs. WT) derived from qRT-PCR data. The right y-axis indicates the log_2_ (fold change) (Mut vs. WT) of mean expression values from RNA-seq data (*n* = 3).; Table S1: Primer sequences of vector construction; Table S2: NcRNAs and mRNAs RT-qPCR primers sequences; Table S3: Dorsal KEGG enrichment analysis of the target genes of DE circRNAs; Table S4: Tail KEGG enrichment analysis of the target genes of DE circRNAs; Table S5: Dorsal KEGG enrichment analysis of the target genes of DE lncRNAs; Table S6: Tail KEGG enrichment analysis of the target genes of DE lncRNAs; Table S7: Dorsal KEGG enrichment analysis of the target genes of DE miRNAs; Table S8: Tail KEGG enrichment analysis of the target genes of DE miRNAs; Table S9: Dorsal KEGG enrichment analysis of the target genes of DE mRNAs; Table S10: Tail KEGG enrichment analysis of the target genes of DE mRNAs; Table S11: Dorsal GSEA of KEGG enrichment analysis; Table S12: Tail GSEA of KEGG enrichment analysis; Table S13: Dorsal Co-expression miRNA- mRNA pairs; Table S14: Tail Co-expression miRNA- mRNA pairs; Table S15: Dorsal Co-expression lncRNA-miRNA-mRNA targeted pairs; Table S16: Tail Co-expression lncRNA-miRNA-mRNA targeted pairs; Table S17: Dorsal Co-expression circRNA-miRNA-mRNA targeted pairs; Table S18: Tail Co-expression circRNA-miRNA-mRNA targeted pairs.
