# Supplementary material for: Lnc007185570.1–pal-miR-3120-3p–ppp3cb Axis Regulating the Osteogenic Differentiation and Intermuscular Bone Formation in Megalobrama amblycephala via the Wnt Signaling Pathway
Source: Biology (Basel). 2026 Aug 5;15(15):1302. doi: 10.3390/biology15151302 (PMC13464817; doi:10.3390/biology15151302)
Supplement: Supplementary file 1 [file biology-15-01302-s001.zip › Supplementary Figure.pdf]

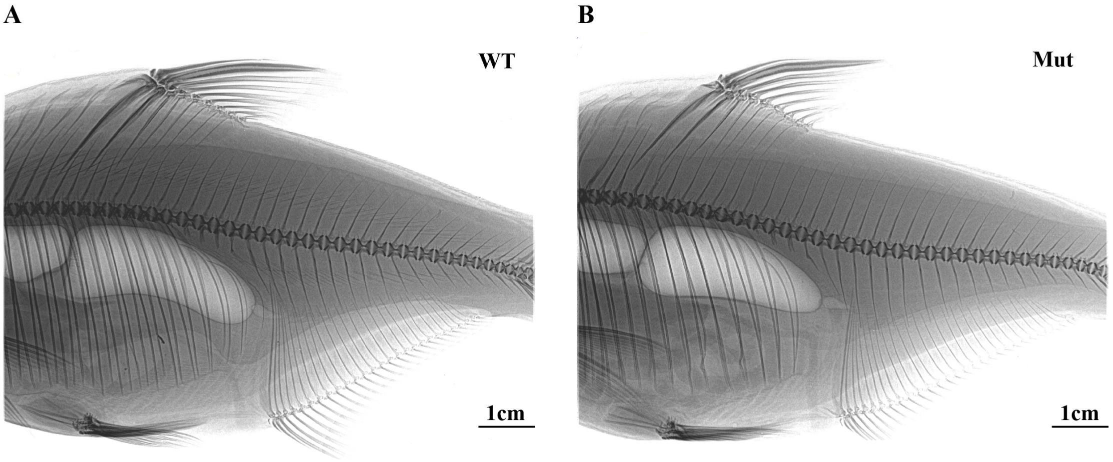

Figure S1. IBs phenotypes in WT and Mut individuals of *M. amblycephala*. (A) X-ray scanning at six-month-old in WT, (B) X-ray scanning at six-month-old in Mut.

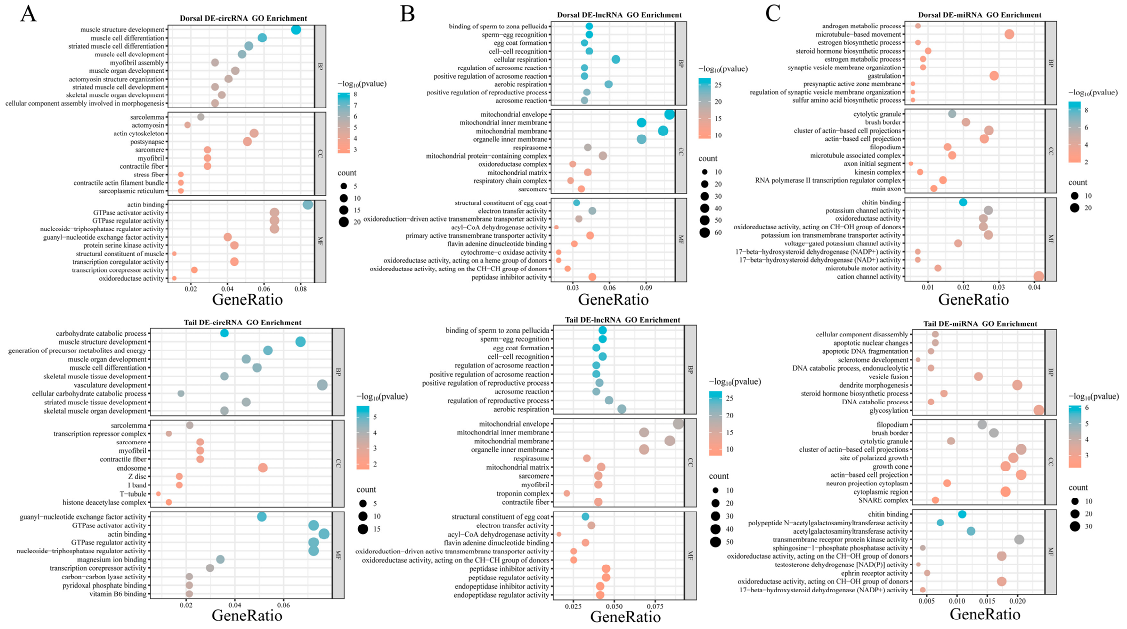

Figure S2. GO enrichment analysis of DE-ncRNAs in dorsal and tail muscle from WT-vs-Mut. (A) GO enrichment analysis of DE-circRNAs target genes in dorsal and tail from WT-vs-Mut, (B) GO enrichment analysis of DE-lncRNAs target genes in dorsal and tail from WT-vs-Mut, (C) GO enrichment analysis of DE-miRNAs target genes in dorsal and tail from WT-vs-Mut.

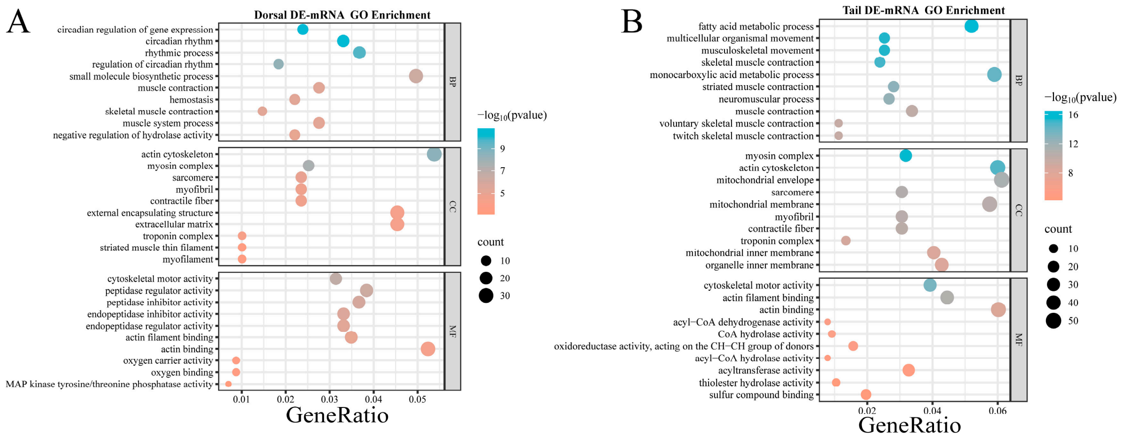

Figure S3. GO enrichment analysis of DE-mRNAs in dorsal and tail muscle from WT-vs-Mut. (A) GO enrichment analysis of DE-mRNAs target genes in dorsal from WT-vs-Mut, (B) GO enrichment analysis of DE-mRNAs target genes in tail from WT-vs-Mut.

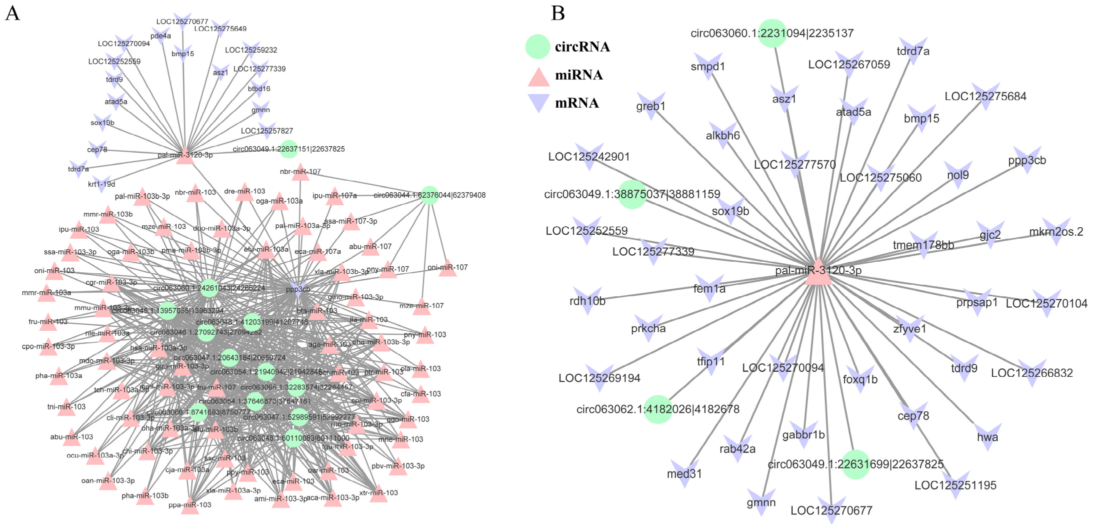

Figure S4. ceRNA network construction in *M. amblycephala* from WT-vs-Mut. (A) circRNA-miRNA-mRNA co-expression network in dorsal, (B) circRNA-miRNA-mRNA co-expression network in tail.

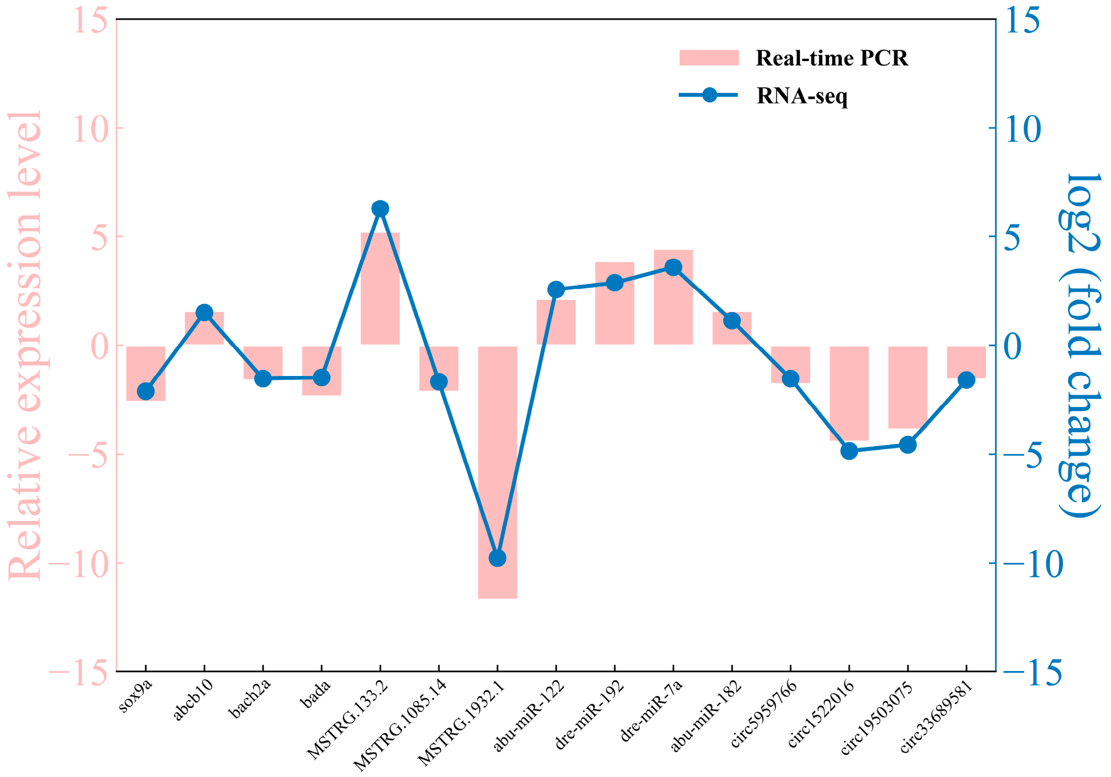

Figure S5. Validation of transcriptome sequencing. * The x-axis represents RNA names, whereas the left y-axis denotes the ratio of mean expression values (Mut vs. WT) derived from qRT-PCR data. The right y-axis indicates the $\log_2$ (fold change) (Mut vs. WT) of mean expression values from RNA-seq data (n=3).
